# Increased de novo glutathione production enhances sexual dysfunctions in rats subjected to paradoxical sleep deprivation

**DOI:** 10.5935/1518-0557.20200070

**Published:** 2021

**Authors:** Leke Jacob Medubi, Nkechi Clara Nwosu, Oluwatoyi Ojuolape Medubi, Olarenaju Ramat Lawal, Cecilia Ama, Taiwo Olabisi Kusemiju, Abraham AA Osinubi

**Affiliations:** 1 Department of Anatomy, Faculty of Basic Medical Sciences, College of Medicine, University of Lagos, Idi-Araba, Lagos, Nigeria; 2 Department of Anatomy, Faculty of Basic Medical Sciences, Gregory University, Uturu, Anabra State, Nigeria; 3 Department of Physiology, Faculty of Basic Medical Sciences, College of Medicine, University of Lagos, Idi-Araba, Lagos, Nigeria

**Keywords:** sleep deprivation, sexual behavior, glutathione

## Abstract

**Objective:**

Poor quality of sexual life has been reported secondary to poor sleep or sleep deprivation. Paradoxical sleep is an integral part of the sleep-wakefulness physiology and prolonged paradoxical sleep deprivation (PSD) may even be fatal. The objective of this investigation was to determine if D-ribose-L-cysteine (RibCys) and zinc (Zn) administration can attenuate the effect of PSD on the sexual function of male rats.

**Methods:**

Following acclimatization, 25 male rats were randomly distributed into five groups of 5 rats each. The PSD, PSD+RibCys, PSD+Zn, PSD+RibCys+Zn, and Control groups were sleep-deprived only, sleep-deprived and given 100mg/kg body-weight of pure RibCys, sleep-deprived and given 10mg/kg body weight Zn, sleep-deprived and given a combination of 100mg/kg of RibCys and 10mg/kg of Zn, and given distilled water without sleep deprivation, respectively. PSD lasted for 20 hours per day for 14 days. Subsequently, the sexual behavioral study was carried out and the animals were sacrificed for biochemical assays.

**Results:**

Analyses of results show that for animals treated with RibCys or Zn, all sexual parameters such as mount frequency and latency, intromission frequency and latency and ejaculation frequency and latency were significantly improved compared with animals subjected to PSD only. This improvement correlates strongly with serum glutathione (GHS) levels.

**Conclusion:**

In summary, riboceine increases circulating GHS, which leads to improved sexual function during sleep deprivation.

## INTRODUCTION

Sleep deprivation is one stressful experience, and it is potentially disruptive to mammalian physical, mental, and emotional coping capacity. Consequently, stress is a physical, mental or emotional demand that is disruptive to natural coping capacity and internal homeostasis of an organism (Mello *et al.*, 2003). Stress reactivity is mediated by physiologically connected distinct anatomical structures, commonly referred to as the hypothalamic-pituitary-adrenal (HPA) Axis (Smith & Vale, 2006). Glucocorticoid levels under the regulation of adrenocorticotrophic hormone (ACTH) is generally accepted as a marker of the intensity of stress reactivity (Burford *et al.*, 2017). Thus, the release of ACTH and corticoids into the bloodstream because of the activation of the HPA axis is a reliable marker of the stress response (Graeff & Zangrossi Junior, 2010). Studies have shown that paradoxical sleep deprivation in rats leads to elevated levels of corticosterone (Andersen *et al.*, 2009; Calegare *et al.*, 2010; Wu *et al.*, 2011; Abd El-Aziz & Mostafa, 2012; Choi *et al.*, 2016).

Male rat’s sexual behavior include penile erection, sexual motivation and mating behavior, and all can be studied while observing the mating behavior of a male rat in direct interaction with a receptive female (Olivier *et al.,* 2011). Typically, a male rat first investigates the female’s face and anogenital region and then approaches the female from the rear, mounts and gives several rapid shallow thrusts and then springs backwards rapidly, and grooms his genitals. They display several mounts and intromissions before ejaculation, which occurs recurrently in a way that a male can achieve several ejaculatory events in a single sexual encounter (Hernandez *et al.*, 2007). After several ejaculations, the male attains sexual satiety and stops mating with the female. Based on this observation, quantification of sexual behaviour normally includes mount frequency, latency, intromission frequency, latency, ejaculation frequency, and post-ejaculatory interval (Olivier *et al.*, 2006).

Male sexual behaviour is partially regulated by the hypothalamic-pituitary-gonadal (HPG) Axis and in virtually all vertebrate species, dependent on testosterone secreted by Leydig cells of the testis (Nyby, 2008). The pattern of testosterone secretion in males is tonic; males are sexually receptive as long as testosterone levels are high (McGinnis & Pfaff, 2012) and there is a reduction in the level of sexual interest during testosterone withdrawal, which is consistent with testosterone being necessary for normal level of sexual interest (Bancroft, 2005).

Circulating testosterone levels are known to increase during sleep and gonadal and sexual functions become impaired secondary to decreased testosterone levels (Abd El-Aziz & Mostafa, 2012). Various studies have shown PSD decrease testosterone levels (Oh *et al.*, 2012; Wu *et al.*, 2011; Arjadi *et al.*, 2014; Alvarenga *et al*., 2015; Choi *et al.*, 2016). Wu *et al.*, (2011) suggested that the decreased serum testosterone levels observed in PSD rats may be the result of 5-HT-related inhibition of testosterone production and decreased testicular expression of STAR protein while, Alvarenga *et al*. (2015) stated that PSD significantly decrease testosterone levels affecting spermatic function in part by interfering in the testicular nitric oxide pathway. It is possible that the two and other mechanisms are involved in testicular steroidogenic dysfunction secondary to sleep deprivation.

Glutathione (GSH) is often referred to as the body’s ‘master antioxidant’ as it is manufactured by the body, but can be increased with certain supplements and dietary modifications. D-Ribose-L-Cysteine (Ribose-Cysteine) is a cysteine analogue developed as a prodrug to support the synthesis of Glutathione (Kader *et al.*, 2014). Zinc is an important trace mineral in male fertility and it is an antioxidant factor that has a profound effect on the level of oxidative stress experienced by the testis (Bao *et al.*, 2010). Increasing zinc levels in males have been shown to boost sperm levels and decrease male infertility while zinc deficiency contributes to the pathogenesis of male reproductive dysfunction (Rajeswari & Swaminathan, 2015).

Oxidative stress is an imbalance between the production of free radicals and the ability of the body to counteract their harmful effects through antioxidants (Mathangi *et al*., 2012), and sleep is a restorative process known to ameliorates oxidative stress and remove oxidants produced during waking (Reimund, 1994). Poor quality of sexual life has been reported secondary to poor sleep or sleep deprivation. Sleep deprivation can cause an irresistible drive to sleep and it is a common type of stress that can have harmful physiological consequences, possibly leading to death in experimental animals (Tufik *et al.*, 2009). Paradoxical sleep is an integral part of the sleep-wakefulness physiology, and prolonged paradoxical sleep deprivation (PSD) may be fatal (Mathangi *et al.*, 2012).

Although studies have shown the effects of zinc therapy on the sexual behaviour of normal rats (Dissanayake *et al.*, 2009; Allouh *et al.*, 2015), no study to the best of our knowledge has been done on paradoxical sleep-deprived rats. Therefore, this study was done to investigate the effects of Zinc and D-ribose-L-cysteine on the sexual behavioral pattern of paradoxical sleep-deprived male rats, with the goal of establishing the relationship between sexual behaviour and redox status in such animals.

## MATERIALS AND METHODS

### Animals

In this study, we used Male and female Sprague-Dawley rats aged 12 weeks weighing about 200±20g. The rats were procured from Jide’s Farm Enterprise, Lagos state and housed at the Animal House of the Department of Anatomy, College of Medicine of the University of Lagos. The animals were allowed to acclimatize for 2 weeks in standard cages, under room temperature, with ratio 1:1 light-dark cycle. Food and water were provided *ad libitum* throughout the study.

### Study Design

At the end of acclimatization, the animals were randomly distributed into 5 groups of 5 rats in each group. The PSD group was subjected to PSD without treatment, while PSD+RibCys, PSD+Zn and PSD+RibCys+Zn groups were, in addition to sleep deprivation, given RibCys, Zn and RibCys plus Zn, respectively. The animals in the control group were kept in their cages and had normal sleep patterns. Zn as zinc sulphate (Bistol Pharmacy Ikeja, Lagos, Nigeria) was given at a dose of 10mg/kg per body weight, while RibCys (Max International, USA) was given at a dose of 100mg/kg per body weight. The drugs were dissolved in 1 mL of normal saline, immediately before oral administration.

### Sleep Deprivation

Sleep deprivation was performed by a modified multiple platform method, a potent method of sleep deprivation (Machado *et al.,* 2004). Briefly, experimental male rats were placed in a custom-made tank containing 6 round platforms of about 6 cm in diameter. The tank was filled with water to about 1 cm below the platform surface. The rats could move around by leaping from one platform to another. The animals were submitted to PSD for 20 hours per day for 14 days, after which the animals were returned to their cages and allowed to sleep for 4 hours. Experimental drugs were administered prior to the sleep deprivation, daily for 14 days.

### Sexual Behaviour Study

The sexual behavioral study was carried out on the 15^th^ day after the sleep deprivation period. Female rats were smeared and the smear was observed under a light microscope, and we used females in their oestrus cycle in the study. Observations were performed under dim red light during the dark phase of the day cycle. Male rats were first placed individually in transparent observation cages for 10 mins, to adapt to the environment. A receptive female was then introduced to each male, by gently dropping him in the cage. Each observation lasted for 30 minutes. We observed and recorded mount frequency and latency, intromission frequency and latency, and ejaculation frequency and latency.

### Blood sampling for hormone measurements

Following slaughter, blood samples were collected by cardiac puncture into plain sample bottles, centrifuged for 15 minutes at 3,000 rpm and the serum was decanted and refrigerated at -20ºC, until assayed for testosterone and corticosterone. Testosterone and cortisol levels were measured by the ELISA method, using the commercial kit (testosterone: Monobind Inc., Lake Forest, CA, USA).

### Statistical Analysis

All parametric values were expressed as means ± standard error of mean (SEM). To determine the difference among various treatment groups, one-way variance analysis was done using Graph Pad Prism 5 (Graphpad Prism Software, Inc., San Diego, CA, USA). Multiple comparisons among various treatment groups were determined by using Bonferroni post hoc comparison test. A *p*-value of<0.05 was considered statistically significant.

## RESULTS

**Figure 1:**
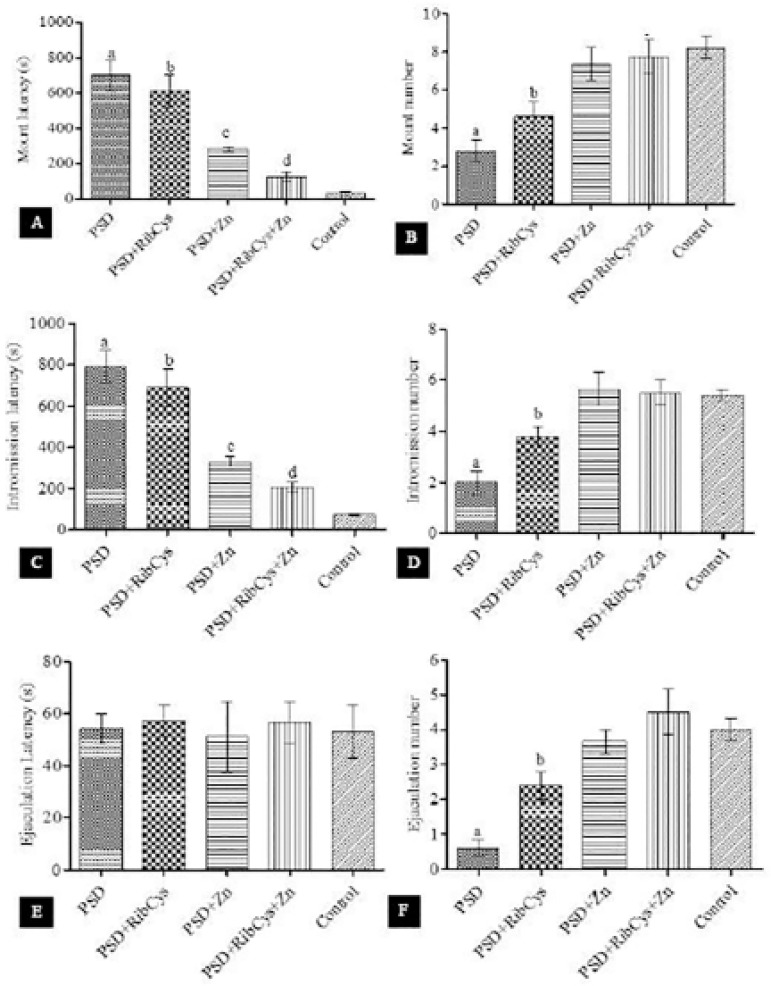
Rat sexual behavioral pattern following sleep deprivation and treatment with RibCys and/or Zn. At a *p*<0.05 level, a indicates significant difference from PSD+RibCys, PSD+Zn, PSD+RibCys+Zn and Control while b indicates a significant difference from PSD+Zn, PSD+RibCys+Zn and Control. c indicates a significant difference from PSD+RibCys+Zn and Control. c while d indicates a significant difference from Controls. PSD=Paradoxical sleep deprivation, RibCys=Riboceine, Zn=Zinc

As shown in Figure 1A, mount latency and intromission latency of the PSD only and PSD + RibCys groups were significantly higher, and the Mount and Intromission frequencies of the PSD only and PSD + RibCys groups were significantly lower when compared with the control group. In panel D, rats in the PSD group showed a statistically significant difference in ejaculation number when compared with the control animals, and although PSD+RibCys is significantly higher than PSD, it is also significantly lower than PSD+Zn, PSD+RibCys+Zn, and control. There was no significant difference in ejaculation latency between the experimental groups.

**Figure 2:**
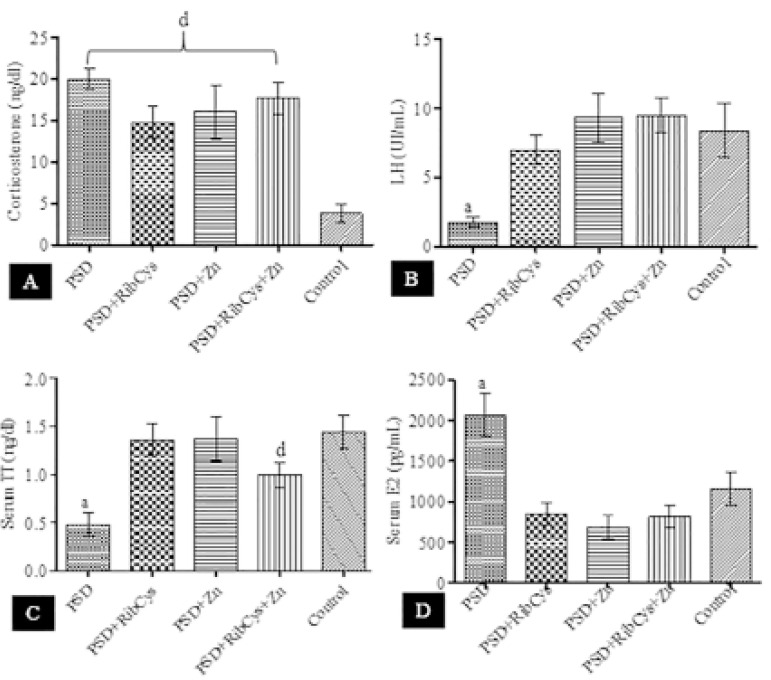
Concentration of serum corticosterone following sleep deprivation and treatment with RibCys and/or Zn.At *p*<0.05 level, a indicates significant differences from PSD, PSD+RibCys, PSD+Zn, PSD+RibCys+Zn and Control while d indicates a significant difference from Control. PSD=Paradoxical sleep deprivation, RibCys=Riboceine, Zn=Zinc.

Figure 2 shows that the serum corticosterone (Figure 2A) level in the PSD, PSD+RibCys, PSD+Zn & PSD+RibCys+Zn groups was significantly higher when compared with the control group. However, there was no significant difference between the treatment groups when compared with the PSD group. In Figure 2B, LH concentration significantly dropped in the PSD group compared with the other groups. No statistical significant differences were observed between the treated groups and their control counterparts. In Figure 2C, serum TT level fell significantly (*p*<0.05) following sleep deprivation compared with either control or treatment groups. However, TT concentration in PSD=RibCys+Zn was significantly lower when compared with the Control group. The level of serum E_2_ rose significantly in the PSD group, compared with any other group as shown in Figure 2D.

**Figure 3:**
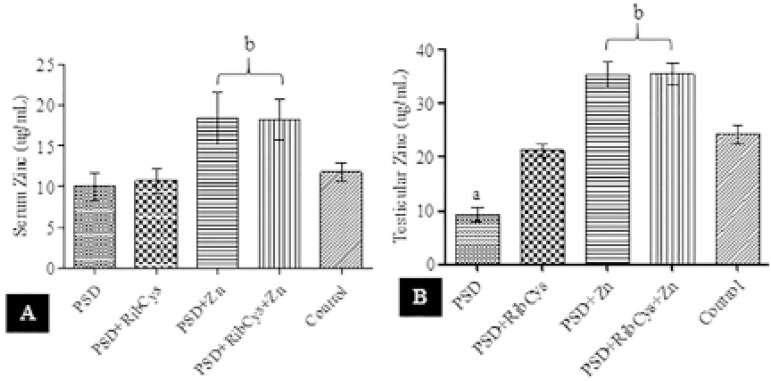
Serum and Testicular Zn concentrations following sleep deprivation and treatment with RibCys and/or Zn.At *p*<0.05 level, a indicates significant differences compared with PSD, PSD+RibCys, PSD+Zn, PSD+RibCys+Zn and Control while b indicates a significant difference from Control. PSD=Paradoxical sleep deprivation, RibCys=Riboceine, Zn=Zinc.

A shown in Figure 3A, Zn concentration levels in PSD and PSD+RibCys were significantly lower compared with PSD+Zn and PSD+RibCys+Zn but not significantly different from the level in Control groups. In Figure 3B, testicular concentration of Zn is significantly lower in PSD compared with PSD+RibCys group, which is not significantly different from that of Control. Both PSD+Zn and PSD+RibCys+Zn had significantly higher Zn concentration compared with any other group.

**Figure 4:**
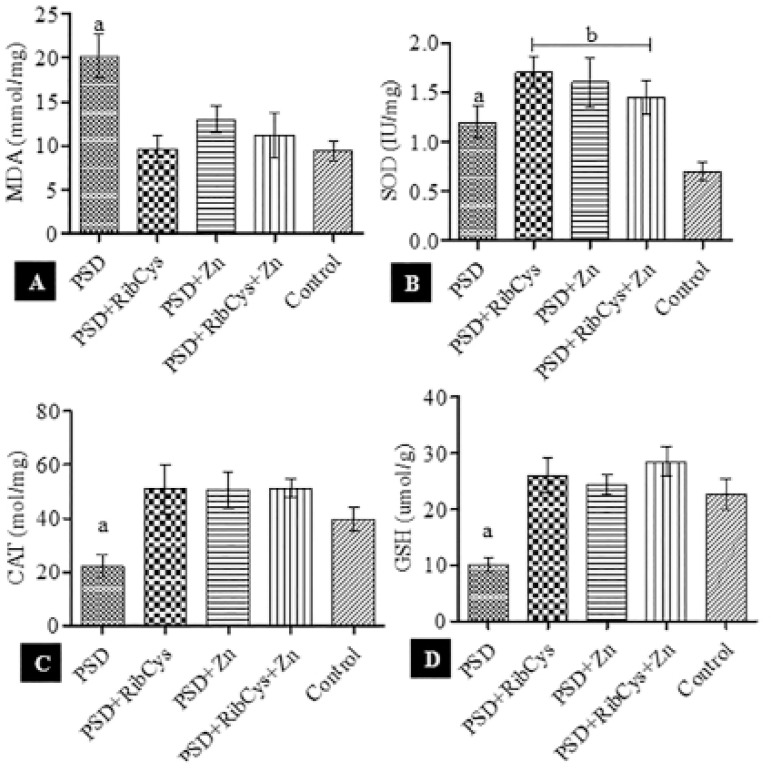
Testicular Redox Status following sleep deprivation and treatment with RibCys and/or Zn. At *p*<0.05 level, a indicates significant differences from PSD+RibCys, PSD+Zn, PSD+RibCys+Zn and Control while b indicates a significant difference from Controls. PSD=Paradoxical sleep deprivation, RibCys=Riboceine, Zn=Zinc

As shown in Figure 4, the MDA level rose significantly in the PSD group compared with the other groups (Figure 4C & D), while CAT and GSH was significantly decreased in the PSD group, compared with the other groups (Figure 4C & D). In Figure 4B, SOD was significantly elevated in all groups compared with controls.

## DISCUSSION

This study was carried out to assess the effects of oral administration of Zn and RibCys on the sexual behaviour of rats that were sleep-deprived for 20 hours per day for a period of 14 consecutive days. Our results indicate a significant increase in mount and intromission latencies, and decrease in mount and intromission frequencies in rats that were subjected to PSD without treatment with the supplements. These findings are consistent with the reports of Alvarenga *et al*. (2009) and Velazquez-Moctezuma *et al.* (1996) but disagree with the results from Ferraz *et al*. (2001). The increase in mount and intromission latencies and decrease in mount and intromission frequencies reflects the low motivation to initiate copulation due to high levels of stress as indicated by elevated glucocorticoid in sleep-deprived rats. This suggests that PSD is stressful enough to cause dysfunctional sexual behaviour. Earlier studies have demonstrated that virtually all stress modalities can significantly interfere with sexual performance (D'Aquila *et al.*, 1994; Retana-Marquez *et al.*, 1996)

It has been shown that the treatment improves sexual competence (Dissanayake *et al.*, 2009). The result of this study showed a non-significant difference in the mount and intromission latencies and mount and intromission frequencies in the PSD group treated with Zinc, at a dose of 10mg/kg of body weight and in the PSD group treated with the combination of Zinc at the stated dose and Riboceine at a dose of 100mg/kg of body weight when compared with the control group, and it also revealed that when compared to the group that was sleep-deprived without any supplementations, the group that was treated with Zinc and the group that was given a combination of both Zinc and Riboceine showed a significant reduction in mount and intromission latencies, and a significant increase in mount and intromission frequencies. This result indicates that the administration of Zinc at 10mg/kg of bodyweight alone or in combination with RibCysat 100mg/kg of body weight, reverses to an extent, the stress caused by PSD on sexual motivation and copulation. However, the group treated with RibCys at 100mg/kg body weight only showed a significant difference in mount and intromission latencies when compared with control and a non-significant difference in mount and intromission latencies when compared with the group that was sleep deprived, only suggesting that Riboceine, at the stated dose, may not be able to reverse the effects of sleep deprivation in sexual performance.

The release of adrenocorticotrophic hormone (ACTH) and corticosterone into the bloodstream, as a result of the activation of the HPA axis is known to be the most characteristic stress response in rodents (Graeff & Zangrossi Junior, 2010). The result in Figure1 showed that all the treatment groups exhibited a significant increase in the level of serum corticosterone regardless of the administration of supplements. This agrees with results from several studies (Andersen *et al.*, 2009; Calegare *et al.*, 2010; Wu *et al.*, 2011; Abd El-Aziz & Mostafa, 2012; Choi *et al.*, 2016). CRH-mediated glucocorticoid secretion during stress is known to inhibit reproductive function (Mello *et al.*, 2003), as evidenced by the increase in mount and intromission latencies, and decrease in the number of mounts and intromissions in the group that was sleep deprived only. However, the administration of Zinc and RibCys was able to counteract the effect of the elevated glucocorticoid on rat sexual performance.

PSD causes changes in the male reproductive system, as it reduces circulating androgens in healthy males, including testosterone (Maia *et al.*, 2011). The result in Figure 2 showed that the PSD group had a significant reduction in the level of serum testosterone when compared with that of the control group, as it has been reported by several studies (Oh *et al.*, 2012; Wu *et al.*, 2011; Arjadi *et al.*, 2014; Alvarenga *et al.*, 2015; Choi *et al.*, 2016). However, Zinc supplementation activates the secretion and action of testosterone (Egwurugwu *et al.*, 2013), as the administration of Zn alone or in combination with RibCys during stress was shown to reverse the drop in testosterone levels as seen by the non-significant difference in the levels of testosterone in the PSD+Zn and PSD+RibCys+Zn groups, when compared with the control group. The PSD group treated with RibCys alone also showed an increase in testosterone levels.

Sexual behaviour in male rats is dependent on testosterone release from the testes, regulated by the hypothalamic-pituitary-gonadal axis, and the pattern of its secretion in males is tonic as males are sexually receptive, as long as testosterone levels are high (McGinnis & Pfaff, 2012). The result from this study agrees with the previous statement that the sleep-deprived group showed a decrease in serum testosterone levels when compared with controls resulted in decreased sexual motivation as evident by the increased mount and intromission latencies.

## CONCLUSION

This investigation reveals that sleep deprivation alters sexual behavior, and reduces male rats’ sexual performance. However, treatment with either RibCyst or Zn or a combination of both attenuates the effects of sleep deprivation on sexual performance through mechanisms that involved lowering testicular oxidative stress level, which in turn protects against PSD-induced suppression of testosterone secretion and/or excessive conversion to estradiol.
